# Advancing Molecular Simulations: Merging Physical Models, Experiments, and AI to Tackle Multiscale Complexity

**DOI:** 10.1021/acs.jpclett.5c00652

**Published:** 2025-04-03

**Authors:** Giorgio Bonollo, Gauthier Trèves, Denis Komarov, Samman Mansoor, Elisabetta Moroni, Giorgio Colombo

**Affiliations:** † Department of Chemistry, University of Pavia, via Taramelli 12, 27100 Pavia, Italy; ‡ National Research Council of Italy (CNR) - Institute of Chemical Sciences and Technologies (SCITEC), via Mario Bianco 9, 20131 Milano, Italy

## Abstract

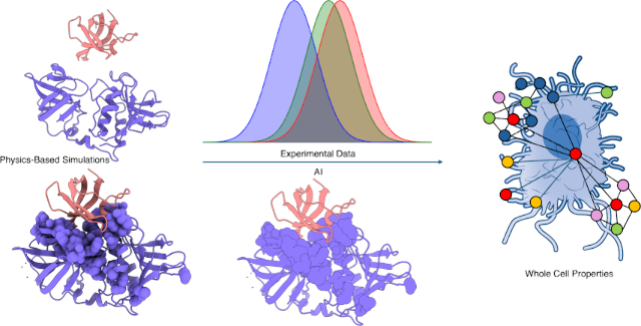

Proteins and protein complexes form adaptable networks that regulate essential biochemical pathways and define cell phenotypes through dynamic mechanisms and interactions. Advances in structural biology and molecular simulations have revealed how protein systems respond to changes in their environments, such as ligand binding, stress conditions, or perturbations like mutations and post-translational modifications, influencing signal transduction and cellular phenotypes. Here, we discuss how computational approaches, ranging from molecular dynamics (MD) simulations to AI-driven methods, are instrumental in studying protein dynamics from isolated molecules to large assemblies. These techniques elucidate conformational landscapes, ligand-binding mechanisms, and protein–protein interactions and are starting to support the construction of multiscale realistic representations of highly complex systems, ranging up to whole cell models. With cryo-electron microscopy, cryo-electron tomography, and AlphaFold accelerating the structural characterization of protein networks, we suggest that integrating AI and Machine Learning with multiscale MD methods will enhance fundamental understating for systems of ever-increasing complexity, usher in exciting possibilities for predictive modeling of the behavior of cell compartments or even whole cells. These advances are indeed transforming biophysics and chemical biology, offering new opportunities to study biomolecular mechanisms at atomic resolution.

Cells are dynamic systems in which complex behavior emerges from a myriad of molecular interactions among proteins and protein complexes. The latter ultimately define the adaptive networks underlying biochemical pathways.

The progress in biochemical and structural approaches sheds light on numerous aspects of the activities of these fundamental molecules and the functional mechanisms of their assemblies.

Some aspects of proteins and protein complexes have proven to be remarkably robust to perturbations, such as the conservation of folds and of 3D supramolecular organizations among homologues from different species.^1,2^ Other aspects are sensitive to even apparently minor disruptions, such as a point mutation, post-translational modifications (PTMs), the binding of ligands, and external factors that push/tweak protein networks into dysfunction.^3−5^

This is strikingly evident in a number of dynamic complexes that control fundamental steps in biochemical pathways. During transcription, for example, complexes form between dynamic and intrinsically disordered activators and similarly disordered coactivator proteins in the transcriptional machinery at precise points in space and time and underpin gene (up)regulation.^6^ Dysregulation of these motifs, and consequently of the structures and mechanisms of their assemblies, is emerging as a critical element for many diseases, including cancer and metabolic disorders.

In the control of protein homeostasis, the core biomolecules responsible for the folding quality control and activation of a plethora of proteins, namely, heat shock proteins Hsp60, Hsp70, and Hsp90, collaborate forming large adaptable complexes which respond to changing cell conditions and environmental stresses.^7−9^ Evidence shows that the interactions among them are rewired in pathologic conditions, in particular, under the effect of disease-associated PTMs.^10^

Finally, in the case of ligand binding, recognition of hormones, peptides, or small molecules to extracellular domains of surface-exposed receptors translates chemical information into intracellular signaling events: while the mechanisms are diverse, ligand binding is often coupled to the induction of novel protein–protein interactions (PPIs), e.g., via dimerization,^11^ which in turn activate signaling cascades.

In all these instances, proteins exist as dynamic ensembles with conformational distributions that may change in response to varying cellular conditions.^12−17^ The variation in the populations of the conformational ensembles caused by some of the above-described perturbations determines the type of structures that are presented to partners for interaction at specific points in time. This is central to allostery, where the redistribution of conformational populations in response to a perturbation causes functional changes at distant sites within the protein.^18^ Through allosteric control of the dynamics of single molecules,^19^ different interactors can thus be selected, generating supramolecular units with different functions and ultimately determining signaling pathways and cell phenotypes.

Several models have been developed to describe “how allostery works” at the molecular level. Linking fundamental understanding of allostery to the structural and spatial organization of supramolecular functional units could allow us to tackle major challenges in biophysics, chemical biology, and systems biology, such as the quantification of the effects of mutations, PTMs, small molecule/metabolite binding. In the wider picture, this would facilitate the identification of new targets and regulatory sites expanding opportunities in drug discovery.

These observations suggest that the dynamic nature of proteins, their complexes and mechanisms of interaction, as well as the Protein–Protein Interaction (PPI) surfaces involved, could be compelling targets for the development of novel chemical tools and drugs.

In this context, it is worth noting that while multiprotein complexes are increasingly recognized as key functional units in the regulation of specific points in biochemical pathways, drug discovery campaigns are still mainly focused on targeting single specific components (often an enzyme). Inhibitors discovered in this way have proved successful in many cases; yet, the complete blockage of a certain activity may indiscriminately impact the entire functional spectrum of the target, resulting in the disruption of all the networks in which it is involved, thus affecting healthy cells as well. In contrast, recent efforts have yielded small molecules that inhibit (or promote) a subset of interchaperone PPIs, and these chemical probes are being used to study networks in their different endogenous conditions and in a range of models.^20−22^

Targeting variable assemblies that regulate task specific networks may indeed open up exciting avenues for chemistry-driven investigations of biology.

To make progress along this path, three main challenges should be met: 1) Understanding dynamic interactions: protein function encompasses numerous intertwined processes, such as enzymatic activities, conformational changes, selection of interacting partners in specific pathways, and functional regulation. Each process involves diverse biomolecular species, in different dynamic configurations and states. 2) Multiscale modeling: protein assemblies operate on multiple scales across both time and space, from atomic-level details to supramolecular structures, with functional properties emerging through cooperative mechanisms and transformations from one scale to another. 3) Decoding functional dynamics: most cellular processes determined by protein assemblies are characterized by the fact that small changes in inputs (encoded, for instance, by ligands) or perturbations (e.g., mutations or post-translational modifications, PTMs) can lead to complex changes in cellular outputs via a modification of the internal dynamics of the complexes.

Meeting these goals would usher in the possibility to construct (virtual) models to simulate, predict, and steer the behavior of protein assemblies, in their endogenous environments (Figure 1).

**Figure 1:**
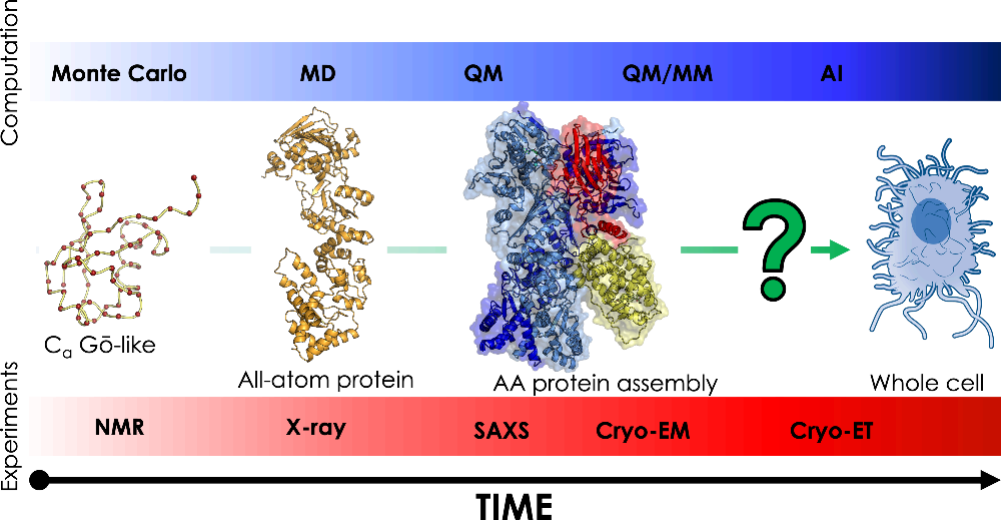
Computational-Experimental evolution of the determination of biological structures starting from small knowledge-based potential and ranging to complex multiprotein assemblies with the perspective of simulating the whole cell. The structures were taken from pdb 1UBQ for the small protein, 2CG9.pdb and 5FWK.pdb for the single protein and complexes representing Hsp90 interactions, respectively, NIAID Visual & Medical Arts for the whole cell representation.

At the present moment, from the experimental point of view, the exponential increase in omics throughput has led to the collection of large (and growing) data sets within and across different cell systems, along with the ability to couple these measurements with systematic perturbations. Computationally, concurrent advances in computing power with the advent of the exascale era^23^ and in AI with the impact of deep learning and generative methods^24,25^ have enhanced our ability to predict structures and learn patterns and processes directly from data without needing explicit rules or human annotation.^4^

There is clearly significant room for chemical-physics-based models to provide precious information and new directions in the study of fundamental biological problems and in the development of novel molecular design methodologies.

In this mini-review, we will try and give a perspective of how molecular simulations at different scales are tackling these problems. We will also explore how the Artificial Intelligence (AI) revolution is reshaping these studies, providing endless possibilities to study complex biological problems at the molecular scale.

## Investigating the Functional Dynamics of Isolated Proteins

The importance and reach of realistic protein models became forcefully evident during the recent Covid pandemic. The possibility to run atomistically detailed simulations of large systems at long lengths allowed the Amaro Group to develop a model for the mechanisms of presentation to human cell receptors of the Receptor Binding Domain (RBD) of the Spike protein from the SARS-CoV-2 Virus.

**Figure 2:**
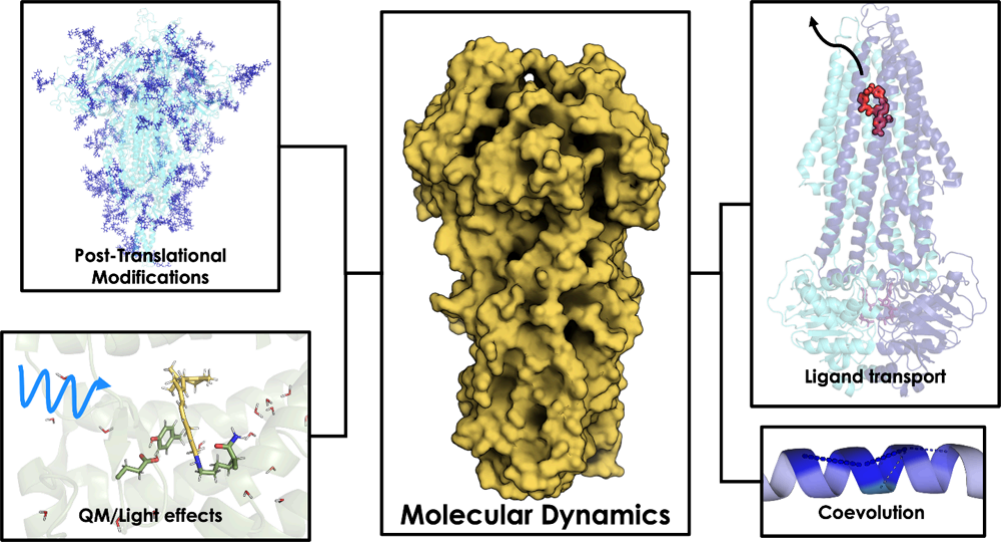
Perspective on different aspects investigated with molecular dynamics simulation: from the effects of glycosylation on Sars-CoV 2 Spike protein, to QM effects of light-responsive phenomena in ipGPCR (structure from pdb 2Z73), passing through the study of Pgp transport cycle (structure from pdb 7ZE5) and coevolutionary studies.

Importantly, the simulations, run on the fully glycosylated form of the protein, revealed essential roles for the sugars in protecting epitopes from being recognized by the host immune system and in modulating the structural dynamics of RBD (Figure 2). These findings were corroborated by biolayer interferometry experiments, which, via mutations of specific residues, showed that deletion of key glycans significantly reduced binding to the human receptor ACE2.^26,27^ Importantly, the authors used the results of their simulations to design stabilized variants of the Spike protein with increased immunogenic potential.^28^

MD simulations have recently been applied to investigate complex basic mechanisms such as the transport of molecules across membranes or the interaction of biological systems with light.

Combining extensive MD with experimental structure resolution methods, Wu et al.^29^ characterized the structure and function of the bacterial heme transporter CydDC. The authors provide detailed insight into the conformational landscape of CydDC during heme binding and occlusion. In particular, simulations show that heme binds laterally from the membrane space to the transmembrane region of CydDC. This is favored by a conformational change that favors an asymmetrical inward-facing CydDC conformation. Simulations, while focusing on a large system, reveal fine details of the mechanisms of heme recognition and transport showing that heme reorientation by 180° is favored by heme propionates first interacting with positively charged residues on the surface and later in the substrate-binding pocket of the transporter (Figure 2).

Gewering investigated P-glycoprotein (Pgp),^30^ a prototypical ATP-binding cassette (ABC) transporter involved in cancer drug resistance and a central mediator of the bioavailability and pharmacokinetics of many drugs.^31^ By covalently attaching a cyclic substrate to select sites on the sequence of Pgp, the authors used cryoEM to determine multiple complex structures in inward- and outward-facing states. Next, they combined these studies with MD simulations to identify conformational changes in transmembrane helix 1 (TM1) as regulators of substrate transport. Overall, the authors trace substrate passage and, importantly, they probe their structural hypotheses via analysis of the effects of mutations designed based on the structural and dynamical understanding of the system (Figure 2).

Fascinating examples of the use of simulations, on different scales, to modulate mechanisms at the cellular scale are provided by photoresponsive proteins. Wijayaratna and collaborators studied Melanopsin, a photopigment belonging to the G Protein-Coupled Receptor (GPCR) family expressed in a subset of intrinsically photosensitive retinal ganglion cells (ipRGCs) and responsible for a variety of processes.^32^ This protein is attractive thanks to its bistability and the possibility to function under low retinal availability, representing an optimal tool for optogenetic investigations. In this work, the authors endeavored to reduce melanopsin’s red light sensitivity, combining in silico structure prediction and automated quantum mechanics/molecular mechanics modeling to predict mutations able to shift melanopsin’s absorption spectrum toward the shorter wavelength region of the visible spectrum (Figure 2).

The designed mutants of mouse melanopsin are shown to trigger macrophage migration by optical activation with blue light, while simultaneously permitting imaging of macrophage migration with red light.

In this framework, it is worth underlining that Olivucci and co-workers also developed a computational protocol for the fast and automated screening of excited-state hybrid quantum mechanics/molecular mechanics (QM/MM) models of the rhodopsin family proteins to be used as fluorescent probes.^33^

The examples discussed above show how different approaches using classical MD simulations, possibly coupled with QM methods, make it possible to investigate realistic models of large proteins in their natural environments.

The trove of data exposed by simulative approaches naturally spurred the evolution of original approaches to highlight the salient determinants of their functionally oriented dynamics. In particular, the identification of functional sites may be relevant for therapeutical and biotechnological applications.

Recently, the integration of sequence, structural, and dynamic data with evolutionary information has started to unveil the mechanisms by which evolution can shape and tune functional dynamics through mutations. Selective pressure, due to adaptation to external stimuli, changes in the environment, and drug action, can induce variations in the kinetic and thermodynamic profiles of protein activities or diversify functions while conserving a common 3D scaffold. Xu and co-workers devised a method that combines coevolutionary analysis with MD simulations to reveal hidden correlations among functional sites.^34^ The method, called DyNoPy, moves beyond the typical structural characterization of coevolving pairs in that it exploits the concept of coevolved dynamical couplings: residue pairs with important functional implications are involved in dynamical interactions that have been preserved during evolution. The authors demonstrate the viability of their approach on SHV-1 and PDC-3, chromosomally encoded β-lactamases linked to antibiotic resistance, showing that the residues that are actually involved in resistance-inducing mutations are connected via coevolution-of-dynamics relationships.

Along these lines, Colizzi and Orozco proposed a way to quantitatively characterize protein-mediated allosteric regulations, combining coevolutionary information, coarse-grained, atomistic simulations, and characterization of the free-energy landscape.^35^ Their approach is applied to investigate adenylyl cyclase (AC) regulation by stimulatory and inhibitory G proteins. A simple on/off mechanism of AC regulation via multiple pathways of information transfer is shaped by the binding of G proteins, which cause a population shift in AC and remodel its free-energy landscape (Figure 2).

Overall, the examples discussed above clearly support the notion that molecular simulations have the potential not only to provide a glimpse into the important motions of proteins but also can be used to reveal relevant insights to tackle fundamental and applicative questions. From the fundamental point of view, the discovery of (hidden) relationships among residues may help further our understanding of the connection between protein sequence and function, as well as help develop models to rationalize their evolutionary trajectories.^36,37^ From the applicative point of view, this molecular-level understanding will help optimize methods to rationally engineer novel proteins with tailored characteristics for specific applications.^28,38^

## From Single Proteins to Large Complexes

Proteins are social molecules and very seldom work in isolation. Rather, they operate in concert with other biomolecules. The study of large realistic complexes is in fact emerging as a novel and important realm of simulations, which also sets the stage for the integration of concepts that range from multiscale and enhanced sampling to AI-driven approaches.

The study of nucleic acid processing machineries is a key example of how the study of large, biologically relevant systems made up of multiple components can provide important chemical insight into a fundamental mechanism and guide the discovery of potential new therapeutic leads.

**Figure 3:**
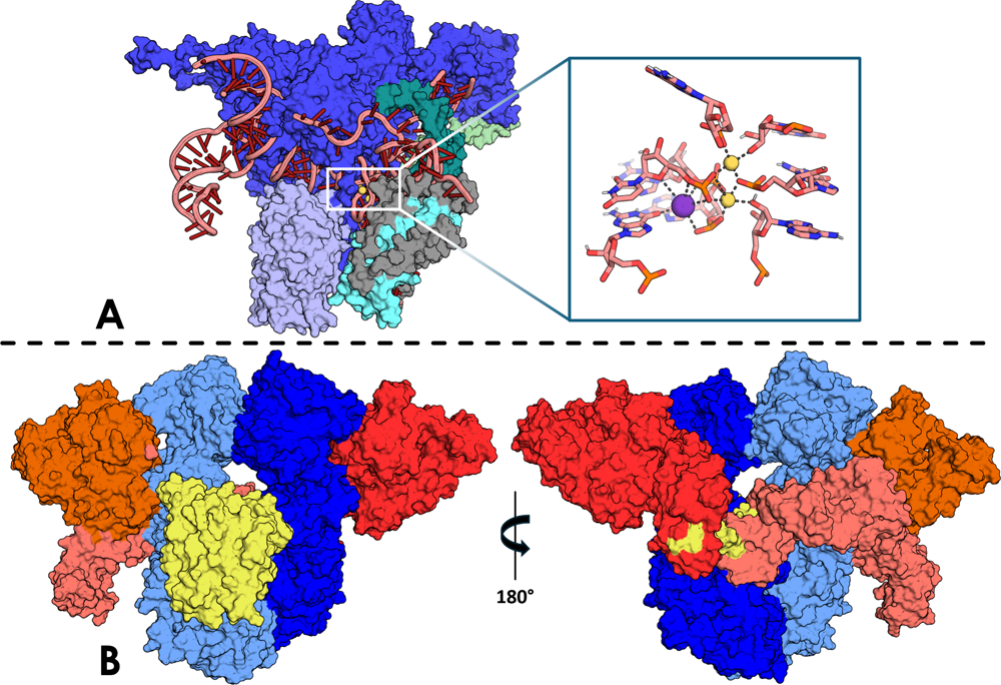
(A) Structure of the spliceosome cellular machinery in complex with RNA. The different partner proteins involved in this cellular machinery are shown as blue, light-blue, cyan, gray, dark green, and green surfaces, while RNA is represented as a pink and red cartoon. In the highlighted box, the interactions of K+ (purple sphere) and Mg^2+^ (yellow spheres) with nucleotides (pink sticks) are shown as gray dashed lines. (B) Structure of the chaperone folding machinery in the loading stage of GR folding cycle (PDB ID: 7KW7). The complex is composed by the two Hsp90 monomers (blue and light-blue surface), Hsp70C (red surface), Hsp70S (orange surface), Hop cochaperone (pink surface), and GR (yellow surface).

The recent availability of a high-resolution structure of the spliceosome, a large cellular machinery composed of both protein and RNA and essential for pre-mRNA splicing in eukaryotes, revealed the presence of a characteristic potassium ion in the active site (Figure 3). Using biased quantum mechanics/molecular mechanics molecular dynamics to elucidate, Aupič et al. showed that this monovalent ion could regulate the kinetics and thermodynamics of the first splicing step through a rigidification of the active site and stabilization of the substrate in the pre- and postcatalytic stages.^39^ This study supports a model in which K+ plays a direct role in directing catalysis in such a large supramolecular assembly. Interestingly, the authors proposed that these mechanisms could be shared by other nucleic acid processing enzymes. Investigations using similar multiscale QM/MM methods were extended to study the role of divalent ions in the Pre-Ribosomal RNA-Processing Machinery.^40^

In this framework, the integration of structural and biochemical approaches with simulation studies favored the discovery of small molecules that recognize self-splicing group II introns, bacterial and organellar ancestors of the nuclear spliceosome.^41^ Identified RNA-binding small molecules were shown to selectively inhibit the two steps of splicing by adopting distinctive poses at different stages of catalysis and by preventing active site conformational changes that are essential for splicing progression (Figure 3).

The study of the process of protein folding quality control has also been impacted by the availability of 3D structures of the multiprotein complexes. In the late stages of folding in the cell, Hsp90 and Hsp70, aided by task-specific cochaperones, process ATP^19^ and become part of a complex network that undergoes extensive compositional and conformational variations. This network drives a chaperoning cycle that orchestrates the structural remodeling of a wide number of substrate proteins, called clients. Using equilibrium and nonequilibrium all-atom MD simulations the fundamental traits of the internal dynamics of the complexes devoted to the loading and maturation of the client Glucocorticoid Receptor (GR) were described.^42^ The loading complex is made up of (GR):Hsp90:Hsp70:cochaperone Hop while the maturation complex is made up of GR:Hsp90:cochaperone p23(Figure 3). Simulations of different nucleotide states of the complexes show how the nucleotide-encoded message is relayed to the client and how the distinct partners of the assemblies cooperate to (pre)organize partially folded GR during Loading and Maturation. Importantly, the presence of different nucleotides in the two chaperones shapes distinct dynamic profiles for the functional interfaces defining the interactions in the complexes and modulate their overall flexibility to facilitate progress along the chaperone cycle. These data support a model where the highly dynamic nature of the assemblies and the conformational heterogeneity of their interactions are key factors in guiding the functions of the assemblies during the chaperoning cycle (Figure 3).

A different process required for aerobic cell life is cellular respiration, which requires the coordinated activity of membrane-bound redox enzymes to shuttle electrons to oxygen and transfer protons across membranes. While there is structural consensus around the fact that these enzymes operate as higher-order supercomplexes, the fine details of their mechanisms still remain elusive. Kaila and co-workers^43^ combined computational studies with biochemical and cryo-electron microscopy experiments to solve the functional dynamics of the 0.7 MDa III_2_IV_2_ obligate supercomplex from *Mycobacterium smegmatis*. From the computational point of view, the authors combined large-scale atomistic molecular dynamics (MD) simulations and hybrid quantum/classical (QM/MM) free energy simulations. The results describe proton and electron pathways responsible for the charge transfer reactions, mechanistic principles of the quinone catalysis, and show how unique molecular adaptations, water molecules, and lipid interactions enable the proton-coupled electron transfer (PCET) reactions. Overall, the results depict a high-resolution picture of supercomplex catalysis through long-range charge transport from its menaquinol oxidation site to the binuclear active site for oxygen reduction.

CRISPR-Cas9 has emerged in the past few years as a revolutionary tool for genome editing. In this large supramolecular assembly, enzymes are combined with nucleic acids to guide the cleavage or the introduction of mutations at desired genome sites via the use of endonuclease Cas9. Clearly, in such a complex system, understanding mechanistic details of the different intertwined processes taking place in the machinery, from DNA cleavage to allosteric control of functions, is a highly challenging task. In this context, the Palermo group used quantum–classical molecular dynamics (QM/MD) and free energy methods to disclose the two-metal-dependent mechanism of phosphodiester bond cleavage in CRISPR-Cas9.^44^ The group went on to provide a molecular picture, inclusive of significant conformational changes in and around the active site, of the various steps of the cleavage. The results could help resolve previous controversial experimental results, which could not fully establish the catalytic role of a conserved H983 and the metal cluster conformation.

Studies of the system were then extended to study the impact of mutations in the Recognition domains and shed light on their allosteric impact on the recognition of DNA.^45^ More recently, the investigations were extended to the mechanisms of xCas9,^46^ an evolved variant which improves specificity for select DNA target sequences while minimizing undesired off-target effects. The authors show that xCas9 introduces flexibility in R1335, providing an entropic advantage for the recognition of specific sequences.

Overall, these studies show how combining different levels of simulations can help expand understanding of CRISPR-Cas, also setting the stage for the design of variants following specific DNA/RNA recognition requirements.

Moving up to even larger scales, Casalino and co-workers^47^ demonstrated how the use of mesoscale simulations of entire virions of the influenza virus, with a full atomistically detailed description of their molecular components, can reveal important points of vulnerability of infection-relevant proteins. The two main surface-exposed targets of influenza are two glycoproteins, hemagglutinin (HA) and neuraminidase (NA), which also represent attractive antigens for the development of immunotherapies. The mesoscale whole-virion model facilitates the understanding of the plasticity of the two glycoproteins in their endogenous environment and reveals three main molecular motions that are thermodynamically and kinetically relevant for the interaction with host receptors or with host immune system molecules: NA head tilting, HA ectodomain tilting, and HA head breathing. Importantly, the motions of HA and NA expose epitope regions that are important for the binding of a novel monoclonal antibody derived from a convalescent human donor. This work describes the previously underappreciated importance of the interplay between the two surface-exposed antigens and may set the stage for the design of new strategies in the development of future vaccines and antivirals.

Interestingly, similar simulative strategies, combined with the introduction of an antibody accessibility score (AAS) that accounts for the steric shielding effect of glycans at the surface of antigenic proteins, were applied to the study of variants of the Spike protein of the SARS-CoV-2 virus.^48^ High values of the AAS are found to efficiently report on the ability of spike protein variants to escape antibody-based immune responses, defining a tool that can be used prospectively to assess the ability of emerging variants to challenge human immune responses.

Large-scale simulations, combined with super-resolution, correlative microscopy, and cryoelectron tomography, have been used to investigate the nuclear entry mechanism of the HIV-1 cone-shaped capsid into the cytoplasm of T cells and macrophages. The capsid enters the nuclear pore complex (NPC) through interactions with phenylalanine-glycine (FG)-repeat nucleoporins (FG-Nups).^49^

The results show that cytosolically bound cyclophilin A is stripped from capsids entering the NPC, and the capsid hexagonal lattice remains largely intact inside and beyond the central channel. The model shows that the scaffold rings of the NPC crack during capsid passage, as the NPC requires widening.

These papers vividly depict the collaborative nature of proteins in different mechanisms, assemblies, and conditions. The common thread is that many components assemble to achieve a shared functional objective. In all cases, the function of the various machineries only emerges when they combine forces. A complete vision of the delicate balance of forces that shape biological pathways can thus be captured when all the components are considered and analyzed at atomistic resolution.

## Moving beyond Protein Assemblies with Computational Microscopes

While for a long time the goal of observing these phenomena at molecular resolution has been out of reach, the developments described above support the possibility of using simulations as a true computational microscope.

The capability of MD simulations to synthesize multiple types of biological data into coherent structural and dynamic models has been exploited by Dommer et al.^50^ to simulate fine aerosols containing whole viral particles. Such aerosols are small particles of diameters in the order of microns that can float in the air for hours and travel into the respiratory tract. The group simulated the aerosol in a realistic biological composition with the inclusion of a full virion model for SARS-CoV-2, lipids, cholesterol, proteins such as albumin, various mono- and divalent salts, mucins, other surfactants, and water. These simulations were shown to provide a novel framework for the study of aerosols: they could allow us to consider varying aerosol composition and size as well as different forms of the virus particle contained, including variants with distinct infectivity profiles.

A notable example of the cooperative combination of different models and scales is represented by the study of the influence of the cell-membrane lipid environment on the gating kinetics of voltage-gated ion-channels.^51^ Carnevale and co-workers propose an approach based on mesoscopic simulations and Ising models of interactions to rationalize the state-dependent channel affinity for different lipid species: this in turn provides a unified explanation for the experimentally observed behaviors of clustering, cooperativity, and hysteresis. By taking into consideration collective behaviors driven by raft formation in critical membranes close to the demixing transition, the model demonstrates that channels support lipid-mediated long-range interactions, activation curve steepening, and long-term memory in ionic currents. Interestingly, the approach suggests a general mechanism for self-organization of biomolecular assemblies in lipid membranes.

In this framework, one of the main directions in the field of simulation appears in the construction of whole-cell models.^52^ Obtaining data at high spatial and dynamic resolution in such complex systems is in fact particularly challenging, but incredible progress is being made in advancing integrative modeling and in understanding the architecture and stoichiometry of cellular components.^53−55^ The minimal cell created by the J. Craig Venter Institute,^56^ a system from a *Mycoplasma* bacterium containing only 493 genes and yet able to independently replicate,^57^ represents an ideal test system for whole cell simulations. The overall dimensions are relatively small size (400 nm in diameter), and its precise composition is known. The Marrink group, using their well-known Martini Coarse-Grained force field,^58^ already put in place efforts to build and simulate this minimal cell (see https://github.com/marrink-lab/Martini_Minimal_Cell).

## New Directions for Molecular Simulations

Advances in computing power, spurred by the transition from using central processing units (CPUs) to graphical processing units (GPUs), have remarkably augmented our possibilities to study the complexity of biological systems at different spatiotemporal scales and with high degrees of resolution. However, several hurdles remain to be overcome to realize the full potential of simulations in designing new biological systems, biotechnological tools, and therapeutics.

The full integration of modeling approaches with advanced structural resolution techniques could provide precious knowledge on how the motions of proteins shape interactions and functions in their natural environments. Understanding how protein networks could be perturbed or modulated and predicting how these perturbations would play out at a cellular scale could be transformative.

A big push in this direction comes from the advent of Artificial Intelligence. Indeed, AI has already been successfully integrated with physics-based simulation techniques to study biomolecular systems. One instance came from the study of large multimolecular systems from the SARS-CoV-2 virus. In this context, Dommer and co-workers^50^ used AI-driven approaches to bridge the gaps in the representations among different scales, thus enabling the study of full aerosol models (containing viral particles, their proteins, mucins) at the atomic/molecular level. Results demonstrate that their integrated data-driven platform provides a new way of exploring the composition, structure, and dynamics of aerosols and aerosolized viruses, while impacting on how simulation methods are developed and applied to unprecedented systems.

One notable example is represented by the introduction of Molecular Dynamics with AI^2^BMD, aimed at combining speed with quantum-chemistry-level accuracy in protein simulations.^59^

**Figure 4:**
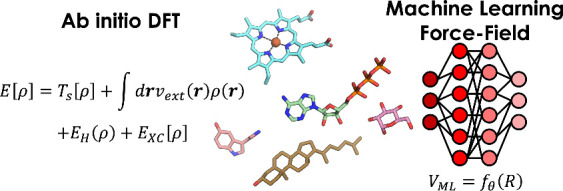
Evolution of the studies of small molecules/peptides: from standard solution of DFT equation in AIMD, to new ML-based techniques that use data from previous simulations to train ML algorithms and speed up QM calculations (structures of some known ligands are reported as example, Heme, ATP, Glucose, Serotonin, and Cholesterol).

While classical MD schemes are based on force calculations using a predefined set of parameters to define the interatomic potential function, in ab initio MD (AIMD), forces are calculated using the potential derived from the electronic structure of molecules. In principle, AIMD can provide accurate characterization of molecules. However, quantum calculations are still expensive, and sampling the energy landscape of a large biomolecule remains out of reach (Figure 4).

The advent of machine learning force fields (MLFFs), trained on data generated at the DFT level and used in classical MD simulations opens up the possibility to obtain highly accurate force calculations at a much lower cost.^60−62^ These methods have been applied to peptides and small proteins but in some cases have been shown to suffer from generalizability and scalability problems. To overcome these limitations, AI^2^BMD uses a model whereby proteins are fragmented into smaller units that are characterized at the DFT level. On this data set, the ViSNet approach is used to derive the potential to calculate the energy and atomic forces for the whole protein under exam. The AI^2^BMD simulation system thus provides a generalizable solution for simulating the MD of proteins. It achieves ab initio accuracy in energy and force calculations. The system was tested on several hundred nanoseconds of dynamics simulations in explicit solvent, demonstrating its ability to efficiently explore the landscapes of peptides and proteins, deriving measurable quantities that accurately matched experimental values, such as ^3^*J* couplings, protein folding/unfolding melting temperature, and free energies and kinetic parameters.

In the context of the structural characterization of protein ensembles, Biomolecular Emulator (BioEmu) is an exciting advancement in the exploration of the conformational spaces of proteins.^63^ Based on a generative deep learning system, BioEmu can generate thousands of statistically independent samples from the protein structure ensemble per hour on a single graphical processing unit. The training of BioEmu, which represents the key challenge in this type of experiment, is performed by combining data ranging from sets of static protein structures to large amounts of MD data, more than 200 ms in total, to experimental measurements of protein stabilities. This generative model is then shown to sample functionally relevant conformational changes, from formation of cryptic pockets to unfolding of specific protein regions and large-scale domain rearrangements.

Finally, it is worth mentioning how *in situ* cryo-electron tomography (cryo-ET) is forcefully emerging as the method that permits study of biomolecular structures in their real native context. Here, the main challenges are represented, on the one hand, by the efficient production of large-scale cryo-ET data sets and, on the other hand, by the association of density maps with the 3D structures of the proteins contained in the biological samples.

Chen and co-workers combined cellular cryo-ET and AlphaFold2 modeling to address these questions. Their endeavor targeted the reconstruction of mammalian sperms. Cryo-ET and subtomogram averaging provided 6.0-A ° reconstructions of microtubules, which allowed them to solve protein tertiary structures. This in turn made it possible to match sperm-specific densities with 21,615 AlphaFold2-predicted protein models of the mouse proteome in an unbiased way. Importantly, the authors were able to identify novel specific microtubule-associated proteins and propose a functional role for these molecules in vivo.^64^

In a similar framework, Kelley et al. used the green alga *Chlamydomonas reinhardtii* as a model organism for *in situ* visualization of cellular processes.^65^ Using cryo-ET data acquisition and processing, they generated a data set of 1829 reconstructed and annotated tomograms, which are provided openly to the community as a resource for method development. The data set includes subtomogram averaging (STA) data for both soluble and membrane-bound complexes ranging in size from >3 MDa to ∼200 kDa, including 80S ribosomes, Rubisco, nucleosomes, microtubules, clathrin, photosystem II, and mitochondrial ATP synthase, with the majority of density maps at subnanometer resolution. These types of workflows and data show that the field can evolve toward visual proteomics.

## Conclusions

The study of protein complexes, their interactions with the environment, and how functional dynamic mechanisms originate is one of the emerging areas of research for computational chemical biology. The development of methods that are both accurate and high-throughput in predicting different conformational states of proteins and protein associations into functional assemblies together with multiscale and AI-driven approaches to simulate whole cell models can have a transformative impact on biochemical/biophysical studies. On the one hand they may provide atomistic descriptions of how perturbations determined by stress factors or by drugs/chemical tools can play out at a cellular level; on the other hand they may be integrated with CryoEM and CryoET studies to enhance the speed with which biochemical systems are characterized. These developments of the field in the coming years promise to usher in exciting new possibilities to investigate biology in atomistic detail shedding light on mechanisms ranging from transient protein–protein interactions to large-scale conformational rearrangements that govern cellular function.

## References

[ref1] Alvarez-Carreno C.; Gupta R. J.; Petrov A. S.; Williams L. D. Creative destruction: New protein folds from old. Proc. Natl. Acad. Sci. U. S. A. 2022, 119 (52), e2207897119 10.1073/pnas.2207897119. 36534803 PMC9907106

[ref2] Rodionov M. A.; Blundell T. L. Sequence and structure conservation in a protein core. Proteins: Struct., Funct., Bioinf. 1998, 33 (3), 358–366. 10.1002/(SICI)1097-0134(19981115)33:3<358::AID-PROT5>3.0.CO;2-0. 9829695

[ref3] Nussinov R.; Tsai C.-J.; Jang H. Signaling in the crowded cell. Curr. Opin. Struct. Biol. 2021, 71, 43–50. 10.1016/j.sbi.2021.05.009. 34218161 PMC8648894

[ref4] Bunne C.; Roohani Y.; Rosen Y.; Gupta A.; Zhang X.; Roed M.; Alexandrov T.; AlQuraishi M.; Brennan P.; Burkhardt D. B.; Califano A.; Cool J.; Dernburg A. F.; Ewing K.; Fox E. B.; Haury M.; Herr A. E.; Horvitz E.; Hsu P. D.; Jain V.; Johnson G. R.; Kalil T.; Kelley D. R.; Kelley S. O.; Kreshuk A.; Mitchison T.; Otte S.; Shendure J.; Sofroniew N. J.; Theis F.; Theodoris C. V.; Upadhyayula S.; Valer M.; Wang B.; Xing E.; Yeung-Levy S.; Zitnik M.; Karaletsos T.; Regev A.; Lundberg E.; Leskovec J.; Quake S. R. How to build the virtual cell with artificial intelligence: Priorities and opportunities. Cell 2024, 187 (25), 7045–7063. 10.1016/j.cell.2024.11.015. 39672099 PMC12148494

[ref5] Paladino A.; Woodford M. R.; Backe S. J.; Sager R. A.; Kancherla P.; Daneshvar M. A.; Chen V. Z.; Bourboulia D.; Ahanin E. F.; Prodromou C.; Bergamaschi G.; Strada A.; Cretich M.; Gori A.; Veronesi M.; Bandiera T.; Vanna R.; Bratslavsky G.; Serapian S. A.; Mollapour M.; Colombo G. Chemical Perturbation of Oncogenic Protein Folding: from the Prediction of Locally Unstable Structures to the Design of Disruptors of Hsp90–Client Interactions. Chemistry - A European Journal 2020, 26 (43), 9459–9465. 10.1002/chem.202000615. 32167602 PMC7415569

[ref6] Pricer R.; Gestwicki J. E.; Mapp A. K. From Fuzzy to Function: The New Frontier of Protein-Protein Interactions. Accounts of chemical research 2017, 50 (3), 584–589. 10.1021/acs.accounts.6b00565. 28945413 PMC5786153

[ref7] Chiosis G.; Digwal C. S.; Trepel J. B.; Neckers L. Structural and functional complexity of HSP90 in cellular homeostasis and disease. Nat. Rev. Mol. Cell Biol. 2023, 24 (11), 797–815. 10.1038/s41580-023-00640-9. 37524848 PMC10592246

[ref8] Schopf F. H.; Biebl M. M.; Buchner J. The HSP90 chaperone machinery. Nat. Rev. Mol. Cell Biol. 2017, 18 (6), 345–360. 10.1038/nrm.2017.20. 28429788

[ref9] Truman A. W.; Bourboulia D.; Mollapour M. Decrypting the chaperone code. J. Biol. Chem. 2021, 296, 100293 10.1016/j.jbc.2021.100293. 33837727 PMC7949055

[ref10] Rodina A.; Wang T.; Yan P. R.; Gomes E. D.; Dunphy M. P. S.; Pillarsetty N.; Koren J.; Gerecitano J. F.; Taldone T.; Zong H. L.; Caldas-Lopes E.; Alpaugh M.; Corben A.; Riolo M.; Beattie B.; Pressl C.; Peter R. I.; Xu C.; Trondl R.; Patel H. J.; Shimizu F.; Bolaender A.; Yang C. H.; Panchal P.; Farooq M. F.; Kishinevsky S.; Modi S.; Lin O.; Chu F. X.; Patil S.; Erdjument-Bromage H.; Zanzonico P.; Hudis C.; Studer L.; Roboz G. J.; Cesarman E.; Cerchietti L.; Levine R.; Melnick A.; Larson S. M.; Lewis J. S.; Guzman M. L.; Chiosis G. The epichaperome is an integrated chaperome network that facilitates tumour survival. Nature 2016, 538 (7625), 397–401. 10.1038/nature19807. 27706135 PMC5283383

[ref11] Tsai C.-J.; Nussinov R. Emerging Allosteric Mechanism of EGFR Activation in Physiological and Pathological Contexts. Biophys. J. 2019, 117 (1), 5–13. 10.1016/j.bpj.2019.05.021. 31202480 PMC6626828

[ref12] Wei G.; Xi W.; Nussinov R.; Ma B. Protein Ensembles: How Does Nature Harness Thermodynamic Fluctuations for Life? The Diverse Functional Roles of Conformational Ensembles in the Cell. Chem. Rev. 2016, 116 (11), 6516–6551. 10.1021/acs.chemrev.5b00562. 26807783 PMC6407618

[ref13] Nussinov R.; Tsai C.-J.; Jang H. Protein ensembles link genotype to phenotype. PLoS computational biology 2019, 15 (6), e1006648–e1006648. 10.1371/journal.pcbi.1006648. 31220071 PMC6586255

[ref14] Hilser V. J. An Ensemble View of Allostery. Science 2010, 327 (5966), 653–654. 10.1126/science.1186121. 20133562 PMC5822688

[ref15] Wrabl J. O.; Gu J.; Liu T.; Schrank T. P.; Whitten S. T.; Hilser V. J. The role of protein conformational fluctuations in allostery, function, and evolution. Biophys. Chem. 2011, 159 (1), 129–141. 10.1016/j.bpc.2011.05.020. 21684672 PMC6583903

[ref16] Morra G.; Genoni A.; Colombo G. Mechanisms of Differential Allosteric Modulation in Homologous Proteins: Insights from the Analysis of Internal Dynamics and Energetics of PDZ Domains. J. Chem. Theory Comput 2014, 10 (12), 5677–5689. 10.1021/ct500326g. 26583250

[ref17] Colombo G.; Daidone I.; Gazit E.; Amadei A.; Di Nola A. Molecular dynamics simulation of the aggregation of the core-recognition motif of the islet amyloid polypeptide in explicit water. Proteins 2005, 59 (3), 519–527. 10.1002/prot.20426. 15778964

[ref18] Wodak S. J.; Paci E.; Dokholyan N. V.; Berezovsky I. N.; Horovitz A.; Li J.; Hilser V. J.; Bahar I.; Karanicolas J.; Stock G.; Hamm P.; Stote R. H.; Eberhardt J.; Chebaro Y.; Dejaegere A.; Cecchini M.; Changeux J.-P.; Bolhuis P. G.; Vreede J.; Faccioli P.; Orioli S.; Ravasio R.; Yan L.; Brito C.; Wyart M.; Gkeka P.; Rivalta I.; Palermo G.; McCammon J. A.; Panecka-Hofman J.; Wade R. C.; Di Pizio A.; Niv M. Y.; Nussinov R.; Tsai C.-J.; Jang H.; Padhorny D.; Kozakov D.; McLeish T. Allostery in Its Many Disguises: From Theory to Applications. Structure (London, England: 1993) 2019, 27 (4), 566–578. 10.1016/j.str.2019.01.003. 30744993 PMC6688844

[ref19] Moroni E.; Agard D. A.; Colombo G. The Structural Asymmetry of Mitochondrial Hsp90 (Trap1) Determines Fine Tuning of Functional Dynamics. J. Chem. Theory Comput. 2018, 14 (2), 1033–1044. 10.1021/acs.jctc.7b00766. 29320629

[ref20] Wong V. Biology in a gray box: targeting the emergent properties of protein complexes: 2011 Yale Chemical Biology Symposium. Yale J. Biol. Med. 2011, 84 (4), 491–495. 22180688 PMC3238318

[ref21] Thompson A. D.; Dugan A.; Gestwicki J. E.; Mapp A. K. Fine-Tuning Multiprotein Complexes Using Small Molecules. ACS Chem. Biol. 2012, 7 (8), 1311–1320. 10.1021/cb300255p. 22725693 PMC3517816

[ref22] Gestwicki J. E. Multi-protein complexes as drug targets. Cell Chemical Biology 2022, 29 (5), 713–715. 10.1016/j.chembiol.2022.05.002. 35594848

[ref23] Beck T. L.; Carloni P.; Asthagiri D. N. All-Atom Biomolecular Simulation in the Exascale Era. J. Chem. Theory Comput. 2024, 20 (5), 1777–1782. 10.1021/acs.jctc.3c01276. 38382017

[ref24] Lappala A. The next revolution in computational simulations: Harnessing AI and quantum computing in molecular dynamics. Curr. Opin. Struct. Biol. 2024, 89, 102919 10.1016/j.sbi.2024.102919. 39306949

[ref25] Muratov E. N.; Bajorath J.; Sheridan R. P.; Tetko I. V.; Filimonov D.; Poroikov V.; Oprea T. I.; Baskin I. I.; Varnek A.; Roitberg A.; Isayev O.; Curtalolo S.; Fourches D.; Cohen Y.; Aspuru-Guzik A.; Winkler D. A.; Agrafiotis D.; Cherkasov A.; Tropsha A. QSAR without borders. Chem. Soc. Rev. 2020, 49 (11), 3525–3564. 10.1039/D0CS00098A. 32356548 PMC8008490

[ref26] Casalino L.; Gaieb Z.; Goldsmith J. A.; Hjorth C. K.; Dommer A. C.; Harbison A. M.; Fogarty C. A.; Barros E. P.; Taylor B. C.; McLellan J. S.; Fadda E.; Amaro R. E. Beyond Shielding: The Roles of Glycans in the SARS-CoV-2 Spike Protein. ACS central science 2020, 6 (10), 1722–1734. 10.1021/acscentsci.0c01056. 33140034 PMC7523240

[ref27] Sztain T.; Ahn S.-H.; Bogetti A. T.; Casalino L.; Goldsmith J. A.; Seitz E.; McCool R. S.; Kearns F. L.; Acosta-Reyes F.; Maji S.; Mashayekhi G.; McCammon J. A.; Ourmazd A.; Frank J.; McLellan J. S.; Chong L. T.; Amaro R. E. A glycan gate controls opening of the SARS-CoV-2 spike protein. Nat. Chem. 2021, 13 (10), 963–968. 10.1038/s41557-021-00758-3. 34413500 PMC8488004

[ref28] Nuqui X.; Casalino L.; Zhou L.; Shehata M.; Wang A.; Tse A. L.; Ojha A. A.; Kearns F. L.; Rosenfeld M. A.; Miller E. H.; Acreman C. M.; Ahn S.-H.; Chandran K.; McLellan J. S.; Amaro R. E. Simulation-driven design of stabilized SARS-CoV-2 spike S2 immunogens. Nat. Commun. 2024, 15 (1), 7370 10.1038/s41467-024-50976-9. 39191724 PMC11350062

[ref29] Wu D.; Mehdipour A. R.; Finke F.; Goojani H. G.; Groh R. R.; Grund T. N.; Reichhart T. M. B.; Zimmermann R.; Welsch S.; Bald D.; Shepherd M.; Hummer G.; Safarian S. Dissecting the conformational complexity and mechanism of a bacterial heme transporter. Nat. Chem. Biol. 2023, 19 (8), 992–1003. 10.1038/s41589-023-01314-5. 37095238 PMC10374445

[ref30] Gewering T.; Waghray D.; Parey K.; Jung H.; Tran N. N. B.; Zapata J.; Zhao P.; Chen H.; Januliene D.; Hummer G.; Urbatsch I.; Moeller A.; Zhang Q. Tracing the substrate translocation mechanism in P-glycoprotein. eLife 2024, 12, RP90174 10.7554/eLife.90174.3. 38259172 PMC10945689

[ref31] Locher K. P. Structure and mechanism of ATP-binding cassette transporters. Philosophical Transactions of the Royal Society B: Biological Sciences 2009, 364 (1514), 239–245. 10.1098/rstb.2008.0125. PMC2674090 18957379

[ref32] Wijayaratna D.; Sacchetta F.; Pedraza-González L.; Fanelli F.; Sugihara T.; Koyanagi M.; Piyawardana S.; Ghotra K.; Thotamune W.; Terakita A.; Olivucci M.; Karunarathne A. In-silico predicted mouse melanopsins with blue spectral shifts deliver efficient subcellular signaling. Cell Communication and Signaling 2024, 22 (1), 394 10.1186/s12964-024-01753-0. 39118111 PMC11312219

[ref33] Pedraza-González L.; Barneschi L.; Marszałek M.; Padula D.; De Vico L.; Olivucci M. Automated QM/MM Screening of Rhodopsin Variants with Enhanced Fluorescence. J. Chem. Theory Comput. 2023, 19 (1), 293–310. 10.1021/acs.jctc.2c00928. 36516450

[ref34] Xu M.; Dantu S. C.; Garnett J. A.; Bonomo R. A.; Pandini A.; Haider S. Functionally Important Residues from Graph Analysis of Coevolved Dynamic couplings; eLife Sciences Publications, Ltd, 2025. 10.7554/eLife.105005 PMC11952748 40153310

[ref35] Colizzi F.; Orozco M. Probing allosteric regulations with coevolution-driven molecular simulations. Science Advances 2021, 7 (37), eabj0786 10.1126/sciadv.abj0786. 34516882 PMC8442858

[ref36] Dindo M.; Pascarelli S.; Chiasserini D.; Grottelli S.; Costantini C.; Uechi G.-I.; Giardina G.; Laurino P.; Cellini B. Structural dynamics shape the fitness window of alanine:glyoxylate aminotransferase. Protein Sci. 2022, 31 (5), e4303 10.1002/pro.4303. 35481644 PMC8996469

[ref37] Tokuriki N.; Tawfik D. S. Protein Dynamism and Evolvability. Science 2009, 324 (5924), 203–207. 10.1126/science.1169375. 19359577

[ref38] Dodani S. C.; Kiss G.; Cahn J. K. B.; Su Y.; Pande V. S.; Arnold F. H. Discovery of a regioselectivity switch in nitrating P450s guided by molecular dynamics simulations and Markov models. Nat. Chem. 2016, 8 (5), 419–425. 10.1038/nchem.2474. 27102675 PMC4843824

[ref39] Aupič J.; Borišek J.; Fica S. M.; Galej W. P.; Magistrato A. Monovalent metal ion binding promotes the first transesterification reaction in the spliceosome. Nat. Commun. 2023, 14 (1), 8482 10.1038/s41467-023-44174-2. 38123540 PMC10733407

[ref40] Borišek J.; Aupič J.; Magistrato A. Third Metal Ion Dictates the Catalytic Activity of the Two-Metal-Ion Pre-Ribosomal RNA-Processing Machinery. Angew. Chem., Int. Ed. 2024, 63 (44), e202405819 10.1002/anie.202405819. 38994644

[ref41] Silvestri I.; Manigrasso J.; Andreani A.; Brindani N.; Mas C.; Reiser J.-B.; Vidossich P.; Martino G.; McCarthy A. A.; De Vivo M.; Marcia M. Targeting the conserved active site of splicing machines with specific and selective small molecule modulators. Nat. Commun. 2024, 15 (1), 4980 10.1038/s41467-024-48697-0. 38898052 PMC11187226

[ref42] Castelli M.; Magni A.; Bonollo G.; Pavoni S.; Frigerio F.; Oliveira A. S. F.; Cinquini F.; Serapian S. A.; Colombo G. Molecular mechanisms of chaperone-directed protein folding: Insights from atomistic simulations. Protein Sci. 2024, 33 (3), e4880 10.1002/pro.4880. PMC10895457 38145386

[ref43] Riepl D.; Gamiz-Hernandez A. P.; Kovalova T.; Król S. M.; Mader S. L.; Sjöstrand D.; Högbom M.; Brzezinski P.; Kaila V. R. I. Long-range charge transfer mechanism of the III2IV2 mycobacterial supercomplex. Nat. Commun. 2024, 15 (1), 5276 10.1038/s41467-024-49628-9. 38902248 PMC11189923

[ref44] Casalino L.; Nierzwicki Ł.; Jinek M.; Palermo G. Catalytic Mechanism of Non-Target DNA Cleavage in CRISPR-Cas9 Revealed by Ab Initio Molecular Dynamics. ACS Catal. 2020, 10 (22), 13596–13605. 10.1021/acscatal.0c03566. 33520346 PMC7842700

[ref45] Skeens E.; Sinha S.; Ahsan M.; D’Ordine A. M.; Jogl G.; Palermo G.; Lisi G. P. High-fidelity, hyper-accurate, and evolved mutants rewire atomic-level communication in CRISPR-Cas9. Science Advances 2024, 10 (10), eadl1045 10.1126/sciadv.adl1045. 38446895 PMC10917355

[ref46] Hossain K. A.; Nierzwicki L.; Orozco M.; Czub J.; Palermo G. Flexibility in PAM recognition expands DNA targeting in xCas9. eLife 2025, 13, RP102538 10.7554/eLife.102538. 39928547 PMC11810106

[ref47] Casalino L.; Seitz C.; Lederhofer J.; Tsybovsky Y.; Wilson I. A.; Kanekiyo M.; Amaro R. E. Breathing and Tilting: Mesoscale Simulations Illuminate Influenza Glycoprotein Vulnerabilities. ACS Central Science 2022, 8 (12), 1646–1663. 10.1021/acscentsci.2c00981. 36589893 PMC9801513

[ref48] von Bülow S.; Sikora M.; Blanc F. E. C.; Covino R.; Hummer G. Antibody accessibility determines location of spike surface mutations in SARS-CoV-2 variants. PLoS Comput. Biol. 2023, 19 (1), e1010822 10.1371/journal.pcbi.1010822. 36693110 PMC9897577

[ref49] Kreysing J. P.; Heidari M.; Zila V.; Cruz-León S.; Obarska-Kosinska A.; Laketa V.; Rohleder L.; Welsch S.; Köfinger J.; Turoňová B.; Hummer G.; Kräusslich H.-G.; Beck M. Passage of the HIV capsid cracks the nuclear pore. Cell 2025, 188, 930 10.1016/j.cell.2024.12.008. 39826544

[ref50] Dommer A.; Casalino L.; Kearns F.; Rosenfeld M.; Wauer N.; Ahn S.-H.; Russo J.; Oliveira S.; Morris C.; Bogetti A.; Trifan A.; Brace A.; Sztain T.; Clyde A.; Ma H.; Chennubhotla C.; Lee H.; Turilli M.; Khalid S.; Tamayo-Mendoza T.; Welborn M.; Christensen A.; Smith D. G. A.; Qiao Z.; Sirumalla S. K.; O’Connor M.; Manby F.; Anandkumar A.; Hardy D.; Phillips J.; Stern A.; Romero J.; Clark D.; Dorrell M.; Maiden T.; Huang L.; McCalpin J.; Woods C.; Gray A.; Williams M.; Barker B.; Rajapaksha H.; Pitts R.; Gibbs T.; Stone J.; Zuckerman D. M.; Mulholland A. J.; Miller T.; Jha S.; Ramanathan A.; Chong L.; Amaro R. E. #COVIDisAirborne: AI-enabled multiscale computational microscopy of delta SARS-CoV-2 in a respiratory aerosol. International Journal of High Performance Computing Applications 2023, 37 (1), 28–44. 10.1177/10943420221128233. 36647365 PMC9527558

[ref51] Suma A.; Sigg D.; Gallagher S.; Gonnella G.; Carnevale V. Ion Channels in Critical Membranes: Clustering, Cooperativity, and Memory Effects. PRX Life 2024, 2 (1), 013007 10.1103/PRXLife.2.013007.

[ref52] Stevens J. A.; Grünewald F.; van Tilburg P. A. M.; König M.; Gilbert B. R.; Brier T. A.; Thornburg Z. R.; Luthey-Schulten Z.; Marrink S. J., Molecular dynamics simulation of an entire cell. Frontiers in Chemistry 2023, 11. 10.3389/fchem.2023.1106495 PMC9889929 36742032

[ref53] Vermaas J. V.; Mayne C. G.; Shinn E.; Tajkhorshid E. Assembly and Analysis of Cell-Scale Membrane Envelopes. J. Chem. Inf. Model. 2022, 62 (3), 602–617. 10.1021/acs.jcim.1c01050. 34910495 PMC8903035

[ref54] Thornburg Z. R.; Bianchi D. M.; Brier T. A.; Gilbert B. R.; Earnest T. M.; Melo M. C. R.; Safronova N.; Sáenz J. P.; Cook A. T.; Wise K. S.; Hutchison C. A.; Smith H. O.; Glass J. I.; Luthey-Schulten Z. Fundamental behaviors emerge from simulations of a living minimal cell. Cell 2022, 185 (2), 345–360. 10.1016/j.cell.2021.12.025. 35063075 PMC9985924

[ref55] Mosalaganti S.; Obarska-Kosinska A.; Siggel M.; Taniguchi R.; Turoňová B.; Zimmerli C. E.; Buczak K.; Schmidt F. H.; Margiotta E.; Mackmull M.-T.; Hagen W. J. H.; Hummer G.; Kosinski J.; Beck M. AI-based structure prediction empowers integrative structural analysis of human nuclear pores. Science 2022, 376 (6598), eabm9506 10.1126/science.abm9506. 35679397

[ref56] Hutchison C. A.; Chuang R.-Y.; Noskov V. N.; Assad-Garcia N.; Deerinck T. J.; Ellisman M. H.; Gill J.; Kannan K.; Karas B. J.; Ma L.; Pelletier J. F.; Qi Z.-Q.; Richter R. A.; Strychalski E. A.; Sun L.; Suzuki Y.; Tsvetanova B.; Wise K. S.; Smith H. O.; Glass J. I.; Merryman C.; Gibson D. G.; Venter J. C. Design and synthesis of a minimal bacterial genome. Science 2016, 351 (6280), aad6253 10.1126/science.aad6253. 27013737

[ref57] Breuer M.; Earnest T. M.; Merryman C.; Wise K. S.; Sun L.; Lynott M. R.; Hutchison C. A.; Smith H. O.; Lapek J. D.; Gonzalez D. J.; de Crécy-Lagard V.; Haas D.; Hanson A. D.; Labhsetwar P.; Glass J. I.; Luthey-Schulten Z. Essential metabolism for a minimal cell. eLife 2019, 8, e36842 10.7554/eLife.36842. 30657448 PMC6609329

[ref58] Souza P. C. T.; Alessandri R.; Barnoud J.; Thallmair S.; Faustino I.; Grünewald F.; Patmanidis I.; Abdizadeh H.; Bruininks B. M. H.; Wassenaar T. A.; Kroon P. C.; Melcr J.; Nieto V.; Corradi V.; Khan H. M.; Domański J.; Javanainen M.; Martinez-Seara H.; Reuter N.; Best R. B.; Vattulainen I.; Monticelli L.; Periole X.; Tieleman D. P.; de Vries A. H.; Marrink S. J. Martini 3: a general purpose force field for coarse-grained molecular dynamics. Nat. Methods 2021, 18 (4), 382–388. 10.1038/s41592-021-01098-3. 33782607 PMC12554258

[ref59] Wang T.; He X.; Li M.; Li Y.; Bi R.; Wang Y.; Cheng C.; Shen X.; Meng J.; Zhang H.; Liu H.; Wang Z.; Li S.; Shao B.; Liu T.-Y. Ab initio characterization of protein molecular dynamics with AI2BMD. Nature 2024, 635 (8040), 1019–1027. 10.1038/s41586-024-08127-z. 39506110 PMC11602711

[ref60] Wang Y.; Wang T.; Li S.; He X.; Li M.; Wang Z.; Zheng N.; Shao B.; Liu T.-Y. Enhancing geometric representations for molecules with equivariant vector-scalar interactive message passing. Nat. Commun. 2024, 15 (1), 313 10.1038/s41467-023-43720-2. 38182565 PMC10770089

[ref61] Smith J. S.; Isayev O.; Roitberg A. E. ANI-1: an extensible neural network potential with DFT accuracy at force field computational cost. Chemical Science 2017, 8 (4), 3192–3203. 10.1039/C6SC05720A. 28507695 PMC5414547

[ref62] Unke O. T.; Stöhr M.; Ganscha S.; Unterthiner T.; Maennel H.; Kashubin S.; Ahlin D.; Gastegger M.; Medrano Sandonas L.; Berryman J. T.; Tkatchenko A.; Müller K.-R. Biomolecular dynamics with machine-learned quantum-mechanical force fields trained on diverse chemical fragments. Science Advances 2024, 10 (14), eadn4397 10.1126/sciadv.adn4397. 38579003 PMC11809612

[ref63] Lewis S.; Hempel T.; Jiménez-Luna J.; Gastegger M.; Xie Y.; Foong A. Y. K.; Satorras V. G.; Abdin O.; Veeling B. S.; Zaporozhets I.; Chen Y.; Yang S.; Schneuing A.; Nigam J.; Barbero F.; Stimper V.; Campbell A.; Yim J.; Lienen M.; Shi Y.; Zheng S.; Schulz H.; Munir U.; Clementi C.; Noé F., Scalable emulation of protein equilibrium ensembles with generative deep learning. bioRxiv 2024. 10.1101/2024.12.05.626885 40638710

[ref64] Chen Z.; Shiozaki M.; Haas K. M.; Skinner W. M.; Zhao S.; Guo C.; Polacco B. J.; Yu Z.; Krogan N. J.; Lishko P. V.; Kaake R. M.; Vale R. D.; Agard D. A. De novo protein identification in mammalian sperm using in situ cryoelectron tomography and AlphaFold2 docking. Cell 2023, 186 (23), 5041–5053. 10.1016/j.cell.2023.09.017. 37865089 PMC10842264

[ref65] Kelley R.; Khavnekar S.; Righetto R. D.; Heebner J.; Obr M.; Zhang X.; Chakraborty S.; Tagiltsev G.; Michael A. K.; van Dorst S.; Waltz F.; McCafferty C. L.; Lamm L.; Zufferey S.; Van der Stappen P.; van den Hoek H.; Wietrzynski W.; Harar P.; Wan W.; Briggs J. A. G.; Plitzko J. M.; Engel B. D.; Kotecha A. Towards community-driven visual proteomics with large-scale cryo-electron tomography of Chlamydomonas reinhardtii. bioRxiv 2024, 630444 10.1101/2024.12.28.630444. 41421337

